# P-866. Epidemiology and Risk Factors for Mortality in Clostridial Bacteremia: A Retrospective Multicenter Observational Study

**DOI:** 10.1093/ofid/ofae631.1057

**Published:** 2025-01-29

**Authors:** Aiko Okazaki, Shu Okugawa, Tatsuya Kobayashi, Miki Kawada, Kyotaro Kawase, Shin Nakayama, Yoshitaka Wakabayashi, Takatoshi Kitazawa, Riko Takezawa, Keita Tatsuno, Saho Koyano, Yoshimi Higurashi, Mahoko Ikeda, Sohei Harada, Takeya Tsutsumi

**Affiliations:** The University of Tokyo Hospital, Bunkyo-ku, Tokyo, Japan; The University of Tokyo Hospital, Bunkyo-ku, Tokyo, Japan; Saitama City Hospital, Saitama, Saitama, Japan; Saitama City Hospital, Saitama, Saitama, Japan; The University of Tokyo Hospital, Bunkyo-ku, Tokyo, Japan; Teikyo University School of Medicine, Itabashi-ku, Tokyo, Japan; Teikyo University School of Medicine, Itabashi-ku, Tokyo, Japan; Teikyo University School of Medicine, Itabashi-ku, Tokyo, Japan; Mitsui memorial hospital, Chiyoda-ku, Tokyo, Japan; Mitsui Memorial Hospital, Chiyoda-ku, Tokyo, Japan; The University of Tokyo Hospital, Bunkyo-ku, Tokyo, Japan; The University of Tokyo Hospital, Bunkyo-ku, Tokyo, Japan; The University of Tokyo Hospital, Bunkyo-ku, Tokyo, Japan; Toho University School of Medicine, Ota-ku, Tokyo, Japan; The University of Tokyo Hospital, Bunkyo-ku, Tokyo, Japan

## Abstract

**Background:**

Clinical studies on clostridial bacteremia are scarce, and the factors associated with mortality have not been well investigated. Furthermore, the distribution of *Clostridium* species varied among studies. To investigate the epidemiology of clostridial bacteremia and the factors associated with mortality, we conducted a study in Japan.

**Figure:**
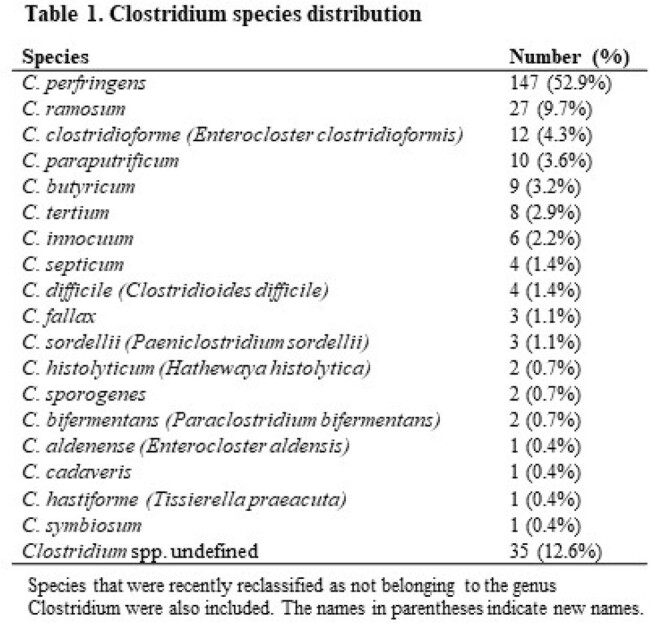

**Methods:**

This multicenter, retrospective, observational study was conducted in four Japanese hospitals. Patients with bacteremia in whom *Clostridium spp*. were detected in blood cultures between 2007 and 2021 were included. Only the first episode of infection was included and blood culture contamination was excluded. Clinical data were collected from the electronic medical records of each hospital. Comparisons between survival and death groups were performed to identify risk factors associated with in-hospital mortality.

**Figure:**
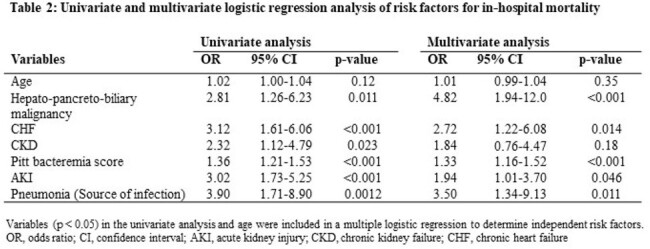

**Results:**

A total of 278 patients were included in the study. *C. perfringens* was the most common species (52.9%), followed by *C. ramosum* (9.7%) (Table 1). The median age of the patients was 77 years, and 61.9% were male. Comorbidities included malignancy (38.1%), diabetes mellitus (29.9%), and gallstones (18.0%). More than 50% of the infections were community-acquired (68.0%). Polymicrobial bacteremia was present in 56.8% of patients. The most common source of infection was the intestinal tract (30.2%) followed by the biliary tract (23.0%). The median Pitt bacteremia score of the patients was two. The in-hospital mortality rate was 25.9%. Of these, 34.7% died within three days of positive blood culture. Mortality rates did not differ significantly between *C. perfringens* and non-*C. perfringens*. The independent risk factors for in-hospital mortality were hepato-pancreato-biliary malignancy, chronic heart failure, Pitt bacteremia score, acute renal failure, and pneumonia as the source of infection (Table 2).

**Conclusion:**

*C. perfringens* was the most common pathogen, but the distribution of other species differed from those reported in other studies. More than one-third of all in-hospital deaths occurred within three days of positive blood culture. To improve prognosis, it is necessary to elucidate the pathogenesis of bacteremia in the early acute phase and develop therapeutic strategies.

**Disclosures:**

**Sohei Harada, MD, PhD**, MSD: Honoraria|Shionogi: Honoraria

